# Comparison of multiplex, ELISA and immunofluorescence for detecting autoantibodies in JIA patients

**DOI:** 10.1186/1546-0096-10-S1-A117

**Published:** 2012-07-13

**Authors:** Paul T Fawcett, Victoria L Maduskuie, Carlos D Rose

**Affiliations:** 1 A I DuPont Hospital for Children, Wilmington, DE, USA

## Purpose

Detection of antinuclear antibodies has traditionally been performed by immunofluorescence (IFA) on fixed culture cells or tissues with reactivity being reported as a fluorescence pattern associated to one or more nuclear antigens. For this study, we compared results from IFA testing with a multiplex antinuclear antibody assay on a panel of patient serum samples previously evaluated by 3 commercial ANA ELISAs.

## Methods

Patient samples: Serum samples from 164 pediatric patients with JIA (82 pauciarticular, 63 polyarticular, 19 systemic) and 20 healthy children (autodonor samples) were tested according to manufacturer’s instructions for all methods. All specimens were stored at -80°C. Testing using 3 ANA ELISAs, IFA and multiplex were performed in batch to minimize freeze thaw cycles. IFA was performed on fixed HEp-2 cell substrate slides with fluorescence pattern and titer determined by an experienced medical technologist. ELISAs included the following antigen mixtures: Sm, RNP, SSA, SSB, Scl-70, JO-1, dsDNA, histones and centromere. The multiplex assay contained substrate beads for individual quantitative detection of antibodies to all of the antigens contained in the ELISAs and qualitative detection of ANA.

## Results

See table 1.

**Table 1:** Evaluation of IFA, multiplex and ELISA antinuclear antibody testing in pediatric JIA patients

		Number testing positive				
		IFA	Multiplex	ELISA		

Types of JIA	# of Samples (n-184)	Hep-2	Microparticle	A.	B.	C.

Pauciarticular	82	62	27 *(3) + **(7)	4 + *(1)	32 + *(5)	52 + *(8)
Polyarticular	63	34	16 *(2) + **(1)	10 + *(4)	30 + *(14)	46 + *(21)
Systemic	19	3	5 + *(4)	1 + *(1)	6 + *(4)	12 + *(12)
All JIA with ≥ 1:160 IFA	164	52	22			
Normals	20	6	1 + **(2)	0 + **(2)	4 + *(1)	5 + *(2)

Numbers in parentheses: * IFA Negative(s) that were Positive. **IFA Positives that were Equivocal

As previously reported, ELISA testing did not correlate with IFA results. Multiplex showed a similar discrepancy with IFA when assessed qualitatively: 48 multiplexed positive JIA versus 98 IFA positives. When higher titer IFA positives (≥ 1:160) were compared with multiplex, findings were similar: 22 multiplex positive versus 52 IFA positive.

## Conclusion

Data obtained from this study indicates that neither ELISA nor multiplex testing, using individually quantifiable antigens, perform the same as IFA to screen pediatric patients with JIA. The lack of agreement between test methods may reflect differences in the array of antigens present in the various assays. Results suggest that both multiplex and ELISA can not replace IFA for routine screening in JIA. However, unlike the ELISAs, the multiplex assay has the advantage of allowing quantitative determination of autoantibodies to each individual nuclear antigen which makes it a superior choice for reflex testing for antibodies to individual nuclear antigens.

## Disclosure

Paul T. Fawcett: None; Victoria L. Maduskuie: None; Carlos D. Rose: None.

